# Comparative Transcriptomic Analysis of Two Apple Cultivars in Response to Dual Cytokinin Applied In Vitro

**DOI:** 10.3390/plants15071001

**Published:** 2026-03-25

**Authors:** Viktor Ambrus, Dóra Farkas, Anita Király, Bianka Tóth, Neama Abdalla, Judit Dobránszki

**Affiliations:** 1 Department of Biotechnology and Microbiology, Faculty of Science and Technology, University of Debrecen, 4032 Debrecen, Hungary; ambrus.viktor@science.unideb.hu; 2 Centre for Agricultural Genomics and Biotechnology, University of Debrecen, 4400 Nyíregyháza, Hungary; gonda-kiraly.anita@agr.unideb.hu (A.K.); toth.bianka@agr.unideb.hu (B.T.); 3 Plant Biotechnology Department, Biotechnology Research Institute, National Research Centre, 33 El Buhouth St., Dokki, Giza 12622, Egypt; na.abdel-aal@nrc.sci.eg

**Keywords:** auxin transport, 6-benzyladenine, cytokinins, cultivar-specific response, cv. Húsvéti rozmaring, cv. McIntosh, hormone metabolism, hormone signaling, kinetin, synergistic effect

## Abstract

The application of dual cytokinins can significantly enhance shoot multiplication rates in specific apple cultivars compared to standard protocols using a single cytokinin. This study presents a comprehensive analysis of shoot multiplication parameters and the underlying transcriptomic response of two distinct apple scion cultivars, cvs. Húsvéti rozmaring and McIntosh, to the simultaneous application of two cytokinins (BA and KIN). Morphological parameters were recorded, followed by comparative RNA-seq analysis and RT-qPCR validation. Our results demonstrate that the BA+KIN treatment induces a unique transcriptomic signature in both cultivars, which cannot be explained by a simple dose–response effect. In cv. McIntosh, 76% of the DEGs were uniquely regulated by the combination, while in cv. Húsvéti rozmaring, although the overlap with single treatments was higher, 17% of the DEGs (representing 1218 genes) were still exclusively activated by the BA+KIN treatment. The fact that the combined treatment recruits specific gene sets and metabolic pathways that remain silent under single BA or KIN applications—regardless of the cultivar—strongly supports a synergistic or non-additive hormonal interaction rather than a response to increased total cytokinin concentration. The dual treatment revealed 3209 DEGs in the inter-cultivar comparison, reflecting distinct strategies: cv. Húsvéti rozmaring achieved high efficiency growth by down-regulating internal hormones, whereas cv. McIntosh exploited intense auxin signaling and hormonal plasticity to maximize bud release. These results prove that distinct molecular pathways can lead to peak performance depending on the apple cultivar.

## 1. Introduction

Currently, micropropagation is the most advanced technical tool for clonal propagation of various plant species, ensuring the appropriate quantity, quality and healthy propagation material. In addition to its commercial importance, micropropagation is also a suitable method for ex situ conservation of genetic resources, in the form of in vitro gene banks [1,2,3]. In vitro propagation of woody plants is a complex process based on meristem-based regeneration, as it ensures genetic stability and minimizes the risk of variability through bypassing callus phase [4]. The development of successful micropropagation protocols requires the coordinated control of many factors, from the appropriate pretreatment and sterilization of explants, to the optimization of nutrient and growth regulator composition, to the fine-tuning of shoot regeneration, proliferation, and rooting [5].

The success of these in vitro protocols fundamentally relies on primary metabolic processes, which play a decisive role in plant growth and development. Primary metabolites are compounds that are essential for the basic life processes and development of the plant [6]. Cytokinins, which are essential plant hormones, play a central role in this regulatory system [7,8]. These hormones are not only involved in the regulation of cell division and shoot formation but also in the delay of senescence and the fine-tuning of stress responses [9,10,11,12,13]. As signaling molecules, they exert local and systemic effects [14,15]. Their biological effects can differ significantly despite their structural similarity, as in the case of kinetin (KIN) and 6-benzyladenine (BA). KIN, as the first identified natural cytokinin [16,17] and BA, a first-generation synthetic cytokinin, are both able to delay senescence processes [7,9,18] but their role in regulating plant immune response and juvenility is based on complex hormonal interactions [19,20,21,22].

Comparison of the effects of KIN and BA, in experimental systems has revealed their functional differences. Hussain et al. [23] emphasized that cytokinins are not only classical growth regulators but also complex signaling nodes that influence the development and stress tolerance of horticultural plants [23]. Bozsó and Barna [24] showed in model plants (*Arabidopsis thaliana* L. and tobacco) that BA induces more powerful morphological, physiological, and transcriptomic changes than KIN. BA effectively stimulated shoot induction but it could involve physiological disorders, which KIN could partially mitigate in *Quercus robur* L. in vitro [25].

The use of plant growth regulators (PGRs) with cytokinin activity is inevitable in the shoot multiplication phase of micropropagation [4,26]. The role of cytokinins in apple cultivars has been addressed by several studies; however, several questions, such as genotype-dependent characteristics or hormonal regulation, still need to be clarified. In the case of apple species (*Malus niedzwetzkyana*, *Malus* × *domestica* Borkh.; from Gala, Royal Gala, and Jonagold grafts), Nurtaza et al. [3] and Sriskandarajah et al. [27] concluded that the combination of KIN and BA could result in a more balanced growth pattern than the application of each hormone alone. Previous classical studies have already shown that KIN can influence the metabolism and development of buds, while BA can have a local effect and undergo metabolic transformation [28]. In his study, Siatka [29] investigated the effect of cytokinin in in vitro callus culture of *Angelica archangelica* L. He analyzed the effect of different concentrations of KIN and BA on biomass growth and anthocyanin accumulation. Based on these results, both cytokinins stimulated growth and pigment formation, with the effect of KIN generally being more moderate compared to BA. At higher concentrations, both cytokinins showed inhibitory effects, reducing cell proliferation and secondary metabolite production, which emphasizes the importance of the concentration-dependent hormonal effect. Tan et al. [30] found that cytokinins play a key role in lateral bud initiation, with higher endogenous cytokinin content in the more branched mutant types and that exogenous cytokinin treatment in the wild type promotes axillary bud growth. If in vitro apple shoots were pretreated by dual cytokinins, e.g., BA+KIN, the rate of adventitious regeneration from leaf explants significantly increased compared to the untreated control [31].

In the current study, two apple scion cultivars with distinct genetic origins were examined. The cv. Húsvéti rozmaring is a historically significant winter apple cultivar from the Carpathian Basin. It was overshadowed in the second half of the 20th century by international cultivars bred for intensive cultivation, but from a gene conservation and pomological perspective, it is still a valuable element of agrobiodiversity [32,33]. In contrast, McIntosh, a cultivar of Canadian origin with international commercial importance, provides a different genetic background for comparison. It played a decisive role in North American apple production for a long time, and is still of high importance from a genetic and breeding perspective and is also well-sold in Europe [34]. These contrasting historical and genetic backgrounds of the two cultivars offer an excellent opportunity to investigate cultivar-dependent transcriptional responses during shoot multiplication.

Despite the documented morphological advantages of combined cytokinin treatments in apple, the molecular mechanisms and the complexity of the underlying gene regulatory networks remain largely unexplored.

The integrated studies of physiological observations and high-throughput transcriptomic data provide means to gain a deeper understanding of these relationships and to fill the knowledge gap. The aim of this study was to investigate how in vitro shoot multiplication parameters and the underlying transcriptomic response of two apple cultivars with different genetic backgrounds (cvs. Húsvéti rozmaring and McIntosh) change when KIN and BA are applied in combination.

We hypothesized that

(1) Dual cytokinin treatment results in more favorable shoot development and growth than either cytokinin applied alone;
(2) The beneficial effect of dual cytokinin application primarily depends on the combined use of the two different cytokinins, BA and KIN, i.e., the effect is rather synergistic than additive;
(3) This synergistic effect, triggered by dual cytokinin application, can be validated and characterized through comparative transcriptomic analysis;
(4) The intensity and molecular background of the response may show cultivar-dependent differences.

## 2. Results

### 2.1. Effect of Cytokinin Content of Shoot Multiplication Medium on the Growth and Development of New Shoots

The cytokinin (CK) content of the shoot multiplication medium significantly influenced the shoot multiplication of both cultivars as well as the growth of shoots, as shown in Table 1. Multiplication rate (shoot number (SN)/explant) was increased when BA or BA+KIN was applied in the shoot multiplication medium compared to the control, CK-free (NCK) medium. However, KIN supplementation failed to increase the shoot number per explant; it was the same as that achieved on CK-free medium, i.e., its value was close to 1.0 in both cultivars. Dual CK supply (BA+KIN) led to a better multiplication rate, i.e., the number of shoots per explant exceeded all other CK treatments. A similar trend can be observed in terms of fresh weight of shoots (SFW). KIN supply did not affect the SFW, BA supply increased it while the dual CK supply (BA+KIN) was the superior for both cultivars. In terms of shoot length (SL), the cultivars responded differently to CK supply. The SL of cv. McIntosh was not affected when the media contained KIN, or BA+KIN. However, BA supply decreased the length of shoots. In the case of cv. Húsvéti rozmaring, KIN increased the length of new shoots compared to those grown on CK-free medium. Single BA and dual CK supply, however, further increased the SL of newly developed shoots, but not differently from each other.

If the responses of cultivars were compared at the same CK supply, the multiplication rate of cv. McIntosh was higher than that of cv. Húsvéti rozmaring at BA supply while at the other CK supplies there were no differences in the SN between the cultivars. Both the SL and the SFW were higher in cv. Húsvéti rozmaring at single BA and dual CK supplies while at KIN supply or on CK-free medium the length and fresh weight of shoots were the same in both cultivars.

### 2.2. Global Assessment of the mRNA Landscape

Transcription profiles were analyzed as the primary indicators of cellular state, representing the initial, highly regulated stage of the gene expression flow. The analysis targeted the expression intensity of 51,804 genes. Three comparisons were performed based on the CK treatments: intra-cultivar assessments across different CK types, and inter-cultivar evaluations conducted at identical CK supplies (Table S1). Volcano plots generated based on CK supply in each cultivar and between cultivars in each CK supply show up- and down-regulated genes for all comparisons (Figure S1).

**Figure 1:**
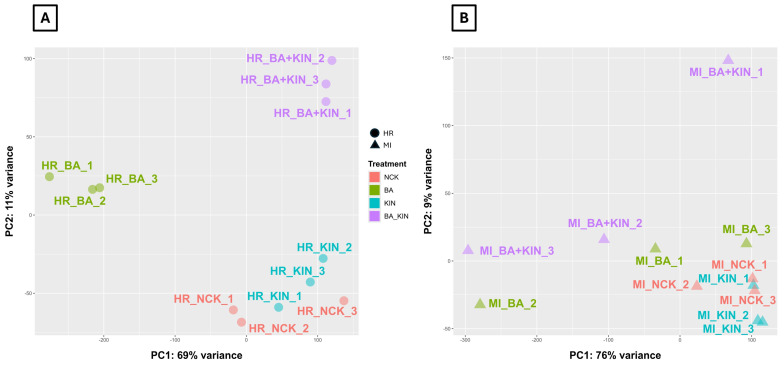
Principal Component Analysis (PCA) of transcriptomic profiles in apple in vitro shoot cultures under various cytokinin treatments for (**A**) Húsvéti rozmaring and (**B**) McIntosh cultivars. Samples were treated with BA, KIN, or dual treatment (BA+KIN), and a CK-free (NCK) treatment was used as a control (BA: 6-benzyladenine; HR: cv. Húsvéti rozmaring; KIN: kinetin; MI: cv. McIntosh; NCK: cytokinin-free).

Principal Component Analysis (PCA) of global gene expression clearly distinguished the different CK treatments in both cultivars. Samples treated with BA (4.5 μM) clustered separately from KIN (4.5 μM) samples, indicating CK-type specific transcriptional responses. The combined treatment (4.5 μM BA + 7.0 μM KIN) formed a distinct cluster that did not lie intermediate between the single treatments, reflecting non-additive transcriptional effects, i.e., demonstrating that even with different CK concentrations applied in single and dual CK treatments, the transcriptional profile is qualitatively distinct from either single treatment (Figure 1).

**Figure 2:**
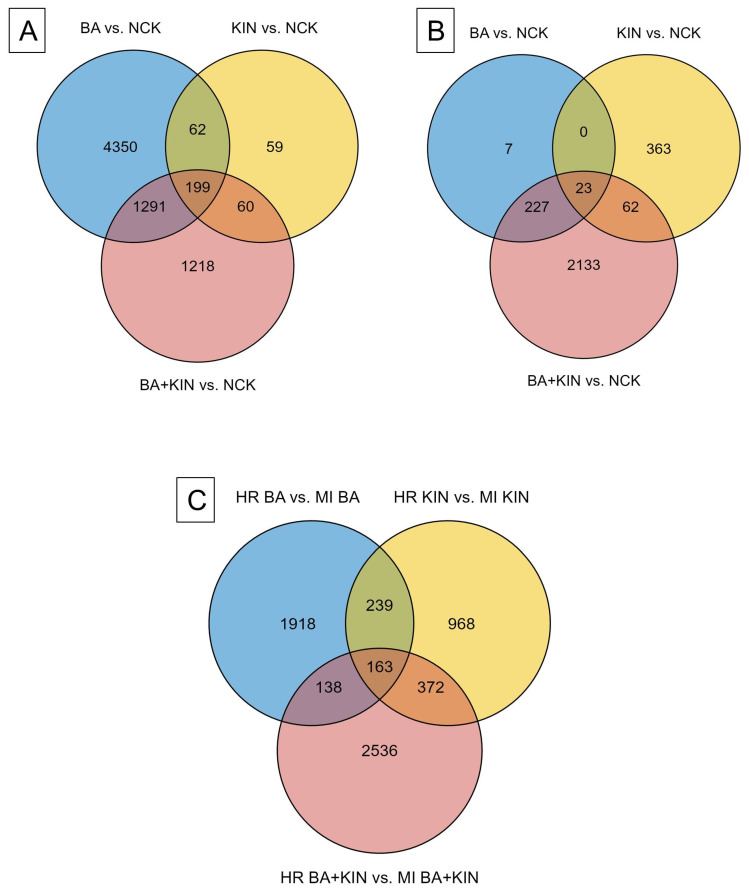
Distribution of significantly differentially expressed genes (DEGs) for (**A**) cv. Húsvéti rozmaring and (**B**) cv. McIntosh shoots following 4 weeks of in vitro cultivation. Comparisons were made between different cytokinin treatments (BA vs. NCK, KIN vs. NCK, and BA+KIN vs. NCK) (intra-cultivar comparisons). (**C**) Inter-cultivar comparisons conducted on identical cytokinin-containing media. (BA: 6-benzyladenine; KIN: kinetin; NCK: cytokinin-free; HR: cv. Húsvéti rozmaring; MI: cv. McIntosh).

During intra-cultivar assessment, BA vs. NCK, KIN vs. NCK, and BA+KIN vs. NCK comparisons were evaluated for cvs. Húsvéti rozmaring (Figure 2A) and McIntosh (Figure 2B). Between-cultivar evaluations (Figure 2C) were performed based on comparisons of both cultivars at BA, KIN, and BA+KIN supplies.

The number of significantly differentially expressed genes (DEGs) were different in the various comparisons. In cv. Húsvéti rozmaring, 5902, 380, and 2768 DEGs were identified in comparisons of BA vs. NCK, KIN vs. NCK, and BA+KIN vs. NCK, respectively. A total of 199 DEGs were identical across all comparisons, while 62 DEGs were identical across the BA vs. NCK and KIN vs. NCK comparisons, 60 DEGs across the KIN vs. NCK and BA+KIN vs. NCK comparisons, and 1291 DEGs across the BA vs. NCK and BA+KIN vs. NCK comparisons (Figure 2A). A total of 7239 DEGs were identified in the cv. Húsvéti rozmaring, whereas only 2815 DEGs were detected in cv. McIntosh, representing a 2.57-fold difference between the two cultivars (Figure 2A,B). In cv. McIntosh, differential expression analysis revealed 257, 448, and 2445 DEGs for the BA vs. NCK, KIN vs. NCK, and BA+KIN vs. NCK comparisons, respectively. Comparative analysis revealed 23 overlapping DEGs across all three conditions. The BA vs. NCK and KIN vs. NCK comparisons shared 227 and 62 DEGs with the combined BA+KIN treatment, respectively. Notably, no common DEGs were identified between the BA vs. NCK and KIN vs. NCK groups (Figure 2B). In the cultivar comparisons, a total of 6334 DEGs were identified. The most extensive transcriptional reorganization was triggered by the combined BA+KIN treatment, yielding 3209 DEGs. In comparison, the individual BA and KIN treatments elicited a more moderate response, representing 76.6% (2458 DEGs) and 54% (1742 DEGs) of the total identified DEGs in the combined supply, respectively (Figure 2C).

Given that our primary objective was to elucidate the transcriptomic impact of dual CK application in both cultivars, a detailed investigation of these synergistic effects was conducted following a global assessment of the expression profiles. To identify the unique transcriptomic signature of the dual CK treatment, we performed a comparative filtering by excluding all DEGs that were also present in the individual BA vs. NCK and KIN vs. NCK comparisons. This allowed us to isolate genes exclusively regulated by the synergistic action of the two CKs. This rigorous filtering process narrowed our focus to 1218 and 2133 unique DEGs for cvs. Húsvéti rozmaring and McIntosh, respectively, alongside 2536 DEGs identified in the inter-cultivar comparison. Our results demonstrate that the BA+KIN treatment induces a unique transcriptomic signature in both cultivars, which cannot be explained by a simple dose–response effect. In McIntosh, 76% of the DEGs (representing 2133 genes) were uniquely regulated by the combination, while in Húsvéti rozmaring, 17% of the DEGs (representing 1218 genes) were exclusively activated by the BA+KIN treatment. The fact that the combined treatment recruits specific gene sets and metabolic pathways that remain silent under single BA or KIN applications—regardless of the cultivar—strongly supports a synergistic or non-additive hormonal interaction rather than a response to increased total CK concentration (Figure 2; Table S1).

### 2.3. Functional Characterization of Biological Processes, Cellular Components, and Molecular Functions Regulated by Dual Cytokinin Application

Functional enrichment analysis of the filtered gene sets revealed GO terms exclusively associated with the dual CK (BA+KIN) treatment, highlighting biological processes, cellular components, molecular functions, and KEGG pathways uniquely triggered by the synergistic action of these hormones (Figure S2; Table S2).

In cv. Húsvéti rozmaring, functional enrichment analysis identified 19 affected biological processes. The most prominent transcriptional changes were concentrated within five key categories, including photosynthesis, light-harvesting, the abscisic acid-activated signaling pathway, and cellular responses to both alcohol and abscisic acid stimuli. Regarding cellular components, DEGs were localized across 20 compartments, with a marked predominance in the plastid and chloroplast thylakoid membranes, as well as photosystem I. Furthermore, 11 molecular functions were annotated, of which six exhibited the highest enrichment levels; these were primarily associated with phosphatase and protein phosphatase regulator/inhibitor activities, alongside isoprenoid and abscisic acid binding. KEGG pathway analysis further complemented these findings, highlighting significant enrichment in porphyrin metabolism and photosynthesis-antenna proteins (Figure S2; Table S2).

In contrast, functional enrichment analysis for cv. McIntosh yielded no significantly overrepresented terms within the biological process or cellular component categories. However, four molecular functions were successfully annotated, primarily centered around transcriptional regulation; these included DNA-binding transcription factor activity (specifically RNA polymerase II-specific), transcription regulator activity, and sequence-specific DNA binding (Figure S2; Table S2).

The inter-cultivar comparison under dual CK supply revealed significant enrichment across 11 biological processes. The most prominent transcriptional responses were concentrated in five categories primarily associated with the regulation of genetic information flow, including DNA-templated transcription, regulation of nucleobase-containing compound metabolic process, regulation of RNA metabolic process, regulation of RNA biosynthetic process, and regulation of DNA-templated transcription. Regarding cellular localization, DEGs were exclusively annotated to the nucleus, while molecular function analysis identified a single enriched category: DNA binding (Figure S2; Table S2).

### 2.4. Differential Gene Expression Analysis of Enzymes and Proteins Related to Hormones

To characterize the molecular response of in vitro apple shoots to dual CK treatment (BA+KIN), a differential gene expression analysis was performed. The resulting transcriptomic profile revealed a profound reprogramming of the hormonal landscape, dominated by a massive shift of the auxin signaling pathway and several antagonistic growth regulators.

**Figure 3:**
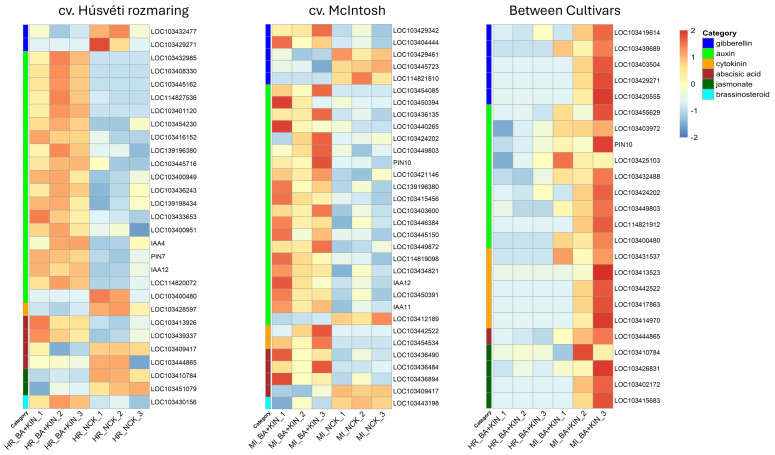
Heat maps showing expression intensity of significantly up- or down-regulated DEGs related to hormonal metabolism and signaling in various cultivars (cvs. Húsvéti rozmaring and McIntosh) and between cultivars at dual cytokinin (BA+KIN) supply. Heat maps generated by pheatmap R package (v1.0.13).

In the case of cv. Húsvéti rozmaring, the most significant transcriptional shift was observed in the auxin-responsive category with 19 identified DEGs (18 up-regulated and one down-regulated). Several genes, particularly the auxin-induced protein 15A-like (AIP15A-like) and two small auxin up-regulated RNA proteins (SAUR21-like variants) (i.e., LOC103432985, LOC103408330, and LOC103445162), exhibited extreme up-regulation with logarithmic fold change (LFC) values ranging from +8.68 to +9.37. Beyond these highly induced transcripts, two auxin-binding proteins (ABP20 and ABP19a) and an auxin response factor (ARF18) also showed increased expression. Within the Aux/IAA family, three genes (IAA3-like, IAA16, and IAA27) were up-regulated, while only IAA1-like showed decreased expression. Finally, auxin transport-related genes were also significantly induced, including the influx carriers; auxin transporter-like protein 2 and 3 (LAX1 and LAX3), and the efflux carrier component 3 (PIN7) (Based on NCBI) (Table 2; Figure 3).

In contrast to the induction of the auxin pathway, the gibberellin (GA) signaling machinery was largely repressed. The expression of a gibberellin receptor (LOC103432477) and a gibberellin 2-oxidase (LOC103429271) gene was significantly decreased, with LFC values of −2.06 and −6.08, respectively. Additionally, evidence of homeostatic feedback in response to the exogenous CK treatment was observed through the down-regulation of a cytokinin riboside gene (LOC103428597, −4.46) (Table 2, Figure 3).

The combined CK treatment also elicited a complex response from pathways associated with stress and senescence. Several abscisic acid (ABA) responsive genes showed divergent expression patterns, with some up-regulated (e.g., LOC103413926, +2.20) while others strongly repressed (e.g., LOC103444865, −3.86). Similarly, the jasmonate (JA) pathway exhibited a consistent downward trend, evidenced by the repression of jasmonate-induced (LOC103410784, −2.51) and methyl jasmonate (LOC103451079, −2.98) related genes. Finally, a brassinosteroid-related gene (LOC103430156) showed an up-regulation of +2.95, suggesting a secondary role for this growth-promoting hormone in the BA+KIN response (Table 2; Figure 3).

The cv. McIntosh transcriptome exhibited an extreme response in genes governing CK homeostasis. The most highly up-regulated gene in the dataset was a cytokinin hydroxylase-like protein (LOC103442522) with an LFC of +10.03, followed closely by cytokinin dehydrogenase 1-like (LOC103454534) at +9.18. This indicates that cv. McIntosh undergoes an intensive metabolic adjustment to process and regulate the levels of exogenous CK within the tissue (Table 2; Figure 3).

Regarding cv. McIntosh, 18 auxin-associated DEGs were identified, including 17 up-regulated and one down-regulated gene. We found that six SAUR variants (SAUR50-like, SAUR12, SAUR12-like, and SAUR51) were up-regulated, along with eight auxin-responsive protein variants (including IAA33, IAA28-like, IAA2-like, IAA13, IAA26, IAA21, IAA26-like, and IAA27). Numerous SAUR genes, such as SAUR50-like (LOC103454085, +9.26) and SAUR12-like (LOC103450394, +7.96), were strongly expressed, alongside multiple Aux/IAA repressors (e.g., IAA33, IAA28-like, and IAA2-like) which showed LFCs ranging from +4.17 to +5.74. Furthermore, genes involved in auxin transport and sensitivity were highly increased in their mRNA levels, including the two efflux carrier components 1b and 1c (LOC103449803 and PIN10) at +5.37 and +5.07. Within this category, only the IAA11-like DEG was found to be down-regulated, with an LFC of −2.08 (Table 2; Figure 3).

The gibberellic acid (GA) pathway in cv. McIntosh showed a complex, biphasic response. While some GA-related genes were significantly up-regulated (e.g., gibberellin 3-beta-dioxygenase 1-like at +6.08 and gibberellin-regulated protein 4 at +4.24), others involved in GA catabolism or responsiveness, such as gibberellin 20-oxidase-like (LOC114821810, −5.20) and gibberellin 2-beta-dioxygenase 1-like (−3.05), were markedly repressed (Table 2; Figure 3).

Similarly, ABA-signaling showed targeted modulation. Genes involved in ABA degradation and sensitivity, such as abscisic acid 8′-hydroxylase 4 (+3.03) and the ABA receptor PYR1-like (+2.50), were induced, while the catabolic gene CYP707A2-like was down-regulated (−3.48). Finally, the brassinosteroid-regulated protein BRU1-like was suppressed (−3.01), further indicating a specific recalibration of the secondary hormonal network in this cultivar (Table 2; Figure 3).

The comparative transcriptomic analysis between the cultivars, both treated with BA+KIN, revealed a consistent and significant down-regulation of genes across several key phytohormone pathways in the Húsvéti rozmaring cultivar relative to McIntosh. All identified DEGs exhibited negative LFC values, ranging from −2.22 to −11.26, indicating a substantial suppression of hormone-related transcripts in cv. Húsvéti rozmaring (Table 2; Figure 3).

A total of five genes related to gibberellin metabolism were significantly down-regulated. This includes multiple isoforms of gibberellin 2-beta-dioxygenase (GA2ox8, GA2ox2-like), which are responsible for deactivating GAs. Notably, gibberellin 20 oxidase 2 (GA20ox2), a key rate-limiting enzyme in active GA biosynthesis, showed a strong down-regulation (−8.88 LFC). The most suppressed gene in this category was gibberellin 3-beta-dioxygenase 1-like (LFC = −10.34), an enzyme that converts inactive precursors into bioactive GAs (Table 2; Figure 3).

The comparison of cv. Húsvéti rozmaring to cv. McIntosh showed the most extensive suppression, all the nine auxin-related genes found are down-regulated. Several auxin efflux carrier components (auxin efflux carrier component 1c, auxin efflux carrier component 1b and auxin efflux carrier component 2-like) were down-regulated; these proteins are essential for maintaining polar auxin transport and distribution. A single small auxin up-regulated RNA variant, SAUR76-like, was also observed among the down-regulated genes. Furthermore, two auxin-responsive proteins (IAA33, and IAA1-like) showed decreased mRNA levels. The strongest down-regulation was observed for auxin-responsive protein IAA1-like (−11.26 LFC). Additionally, we identified an auxin response factor (ARF2-like) that exhibited significant down-regulation, with a LFC of −6.00 (Table 2; Figure 3).

Despite that, both cultivars received the same CK treatment, cv. Húsvéti rozmaring showed a reduction in transcripts for endogenous CK metabolism compared to cv. McIntosh. Five genes were affected, including cytokinin dehydrogenase 3-like (involved in degradation) and multiple cytokinin hydroxylase-like genes. The suppression of cytokinin riboside 5′-monophosphate phosphohydrolase (−10.94 LFC) suggests a reduction in the activation of CK precursors in the Húsvéti rozmaring cultivar (Table 2; Figure 3).

Additionally, genes related to ABA and JA were down-regulated. Abscisic acid 8′-hydroxylase CYP707A2, the primary enzyme for ABA catabolism, was suppressed (−4.83 LFC). The JA pathway featured down-regulation of jasmonate-induced oxygenase 1-like and several methyl jasmonate esterases (up to −10.65 LFC), which regulate the levels of the active JA signal (Table 2; Figure 3).

### 2.5. Validation of mRNA-Seq Data with RT-qPCR

GAPDH was utilized as the internal housekeeping gene for normalization, as its expression remained constant across all experimental groups, ensuring the accuracy of the comparative analysis.

The expression patterns observed in the RT-qPCR analysis were highly consistent with the mRNA-seq data, displaying identical trends in both up- and down-regulation. A Pearson correlation analysis revealed a coefficient of R = 0.96 (*p* = 0.0029) and R = 0.95 (*p* = 0.013) for cv. Húsvéti rozmaring and cv. McIntosh respectively, indicating a strong positive correlation between the transcriptomic and quantitative PCR LFC values (Table S3).

## 3. Discussion

The morphological evaluation of cvs. Húsvéti rozmaring and McIntosh shoot growth and development confirmed a clear synergistic effect between BA and KIN, as the dual CK supply proved superior to all other treatments in promoting both axillary shoot multiplication and biomass accumulation (Table 1). While KIN as a sole CK source failed to surpass the multiplication rate of the CK-free control (NCK), its presence in the BA+KIN combination significantly enhanced the multiplicative effect of BA. This suggests that while BA serves as the primary trigger for the loss of apical dominance and subsequent axillary bud outgrowth, KIN acts as a critical co-factor that optimizes the multiplication process. This synergy is particularly valuable for improving the multiplication rate (SN) while simultaneously increasing the fresh weight (SFW) of newly developed in vitro shoots. The cultivars, however, exhibited divergent growth strategies regarding shoot architecture. In cv. McIntosh, the high multiplication rate induced by BA treatments was accompanied by a significant reduction in shoot length (SL). This is a common phenomenon in in vitro cultures, where vigorous lateral budding often occurs at the expense of longitudinal growth [4,26]. Conversely, cv. Húsvéti rozmaring showed remarkable phenotypic robustness; the dual CK supply not only maximized the number of shoots but also maintained superior shoot length and biomass. This indicates that cv. Húsvéti rozmaring possesses a higher capacity to balance cell division with cell expansion. These morphological findings highlight that for apple cultivars with differing growth habits, the synergistic application of BA and KIN is beneficial from a micropropagation point of view. This dual CK strategy ensures an optimal balance between high-intensity shoot number and overall shoot quality, providing a more efficient micropropagation system.

In both the single and dual CK treatments, we deliberately selected concentrations that fall within the range commonly applied for shoot multiplication in apple, representing the minimal levels expected to induce a reliable shoot proliferation response [4]. Thus, the experimental design aimed to avoid supra-optimal effects and instead reflect biologically relevant CK activity during the shoot multiplication phase of micropropagation. Despite the slightly higher total CK content in the combined treatment (BA+KIN), our data clearly indicate that transcriptional divergence was driven by the CK type rather than the CK quantity. BA and KIN, applied single and at identical concentrations (4.5 μM), already induced markedly distinct global expression profiles, as evidenced by their clear separation in the PCA plots (Figure 1) and the presence of CK-specific DEG sets (Figure 2; Table S1). Therefore, qualitative differences in signaling occurred independently of dosage variation. Furthermore, the combined treatment did not produce a linear or additive transcriptional shift relative to the single CK treatments; instead, it formed a distinct expression cluster and included combination-specific DEGs absent from either individual treatment. Such non-additive behavior is incompatible with a simple CK concentration-dependent response and instead supports differential receptor interaction and downstream signaling integration. Therefore, these findings demonstrate that CK-type specific signaling, rather than total CK load, is the primary determinant of transcriptomic reprogramming in apple.

The transcriptomic profiling of cvs. Húsvéti rozmaring and McIntosh revealed fundamentally different strategies in their molecular responses to exogenous CK applications (Figure 2). The substantial difference in the number of DEGs (7239 vs. 2815) correlates with the distinct genetic and breeding histories of the two cultivars. The cv. Húsvéti rozmaring is a traditional cultivar from the Carpathian Basin while cv. McIntosh is an intensive commercial cultivar of Canadian origin. The 2.57-fold higher number of DEGs in cv. Húsvéti rozmaring compared to cv. McIntosh (Figure 1A,B) suggests a heightened sensitivity or a more complex regulatory network in the former cultivar under CK-mediated shoot multiplication conditions. A key finding of this study is the contrasting impact of individual CK types on the two genotypes. In cv. Húsvéti rozmaring, BA appeared to be the primary driver of transcriptional changes, inducing over 15 times more DEGs than KIN (Figure 2A). In contrast, cv. McIntosh exhibited a more balanced, albeit significantly more moderate, response to these individual stimuli. The lack of common DEGs between the BA vs. NCK and KIN vs. NCK comparisons in cv. McIntosh indicates that these two CKs may trigger independent signaling pathways or target different gene sets in this cultivar (Figure 2B), whereas in cv. Húsvéti rozmaring, a substantial overlap suggests a more unified CK-response mechanism (Figure 2A). The results show the cultivar-specific sensitivity of apple cultivars to BA and KIN. Our findings align with previous transcriptomic studies of Bozsó and Barna [24] in Arabidopsis and tobacco, confirming that BA and KIN exert fundamentally different effects on the transcriptome, which explains why these CKs induce distinct morphological responses even in diverse experimental systems, such as in vitro apple cultures. The most profound transcriptional reorganization across both cultivars and in the inter-cultivar comparisons was elicited by the combined BA+KIN treatment. In cv. Húsvéti rozmaring, while the individual BA treatment elicited the highest absolute number of DEGs, the combined BA+KIN supply triggered an extensive transcriptional shift. This synergistic effect was evidenced by the identification of 1218 unique DEGs that were exclusively regulated under the dual CK treatment, representing a specialized response not observed under single CK applications (Figure 2A). The synergistic effect is most evident in cv. McIntosh, as well, where the dual application induced 2445 DEGs—a nearly 10-fold increase compared to BA alone and a 5-fold increase compared to KIN alone (Figure 2B). In the inter-cultivar comparison, the fact that the BA+KIN supply triggered the highest number of DEGs (3209) (Figure 2C) further underscores that the combination of these CKs creates a unique physiological state that cannot be achieved by individual applications. This synergy likely reflects a crosstalk between different CK-signaling components, leading to the activation of downstream metabolic and developmental pathways that are crucial for in vitro shoot organogenesis.

The identification of 199 and 23 DEGs, which were identical across all treatments in cvs. Húsvéti rozmaring and McIntosh, respectively (Figure 2A,B), points toward a small but potentially vital set of genes responsible for general CK sensing. However, the high number of unique DEGs found exclusively in the BA+KIN vs. NCK comparisons (after filtering out individual effects; Figure 2) suggests that dual CK application does not merely sum the effects of BA and KIN but rather recruits a specific set of genes. These genes are likely responsible for the improved multiplication rates observed in dual CK systems in both apple cultivars (Table 1).

The functional annotation of filtered DEGs (Figure S2) provides a molecular explanation for the observed synergistic effects of dual CK (BA+KIN) application. Our results demonstrate that the synergy not only amplifies general CK responses but also triggers cultivar-specific physiological pathways that are absent under single-hormone treatments. In cv. Húsvéti rozmaring, the dual CK supply appears to act as a potent regulator of the photosynthetic apparatus. The exclusive enrichment of GO terms related to light-harvesting complexes, thylakoid membrane components, and photosystem I, integrated with KEGG findings on porphyrin metabolism, suggests that the BA+KIN combination specifically promotes chloroplast functional integrity and photosynthetic efficiency. This robust metabolic response is further characterized by a complex hormonal crosstalk. The activation of abscisic acid (ABA)-related signaling and phosphatase inhibitor activities indicates that cv. Húsvéti rozmaring utilizes a fine-tuned regulatory network to balance growth-promoting CK signals with developmental ABA pathways. This response suggests a high level of physiological plasticity in this cultivar when transitioning to dual CK multiplication medium. In stark contrast, cv. McIntosh exhibited a much more restricted transcriptomic response. The absence of significantly enriched biological processes or cellular components—despite the identification of over 2000 unique DEGs—suggests that the dual CK stimulus in this cultivar primarily occurs in the pre-metabolic stage. The enrichment of DNA-binding transcription factor activities and RNA polymerase II-specific regulators implies that cv. McIntosh focused its cellular energy on the upstream recalibration of its transcriptional machinery. While cv. Húsvéti rozmaring had already translated the hormonal signal into downstream metabolic changes (e.g., photosynthesis), cv. McIntosh appeared to remain in a “preparatory” regulatory phase, which may explain its different in vitro growth characteristics as well. The inter-cultivar comparison further confirmed that the core difference between these two genotypes lies in the nucleus and the regulation of genetic information flow. The significant enrichment of RNA biosynthetic and DNA-templated transcription processes underscores that the two cultivars possess fundamentally different “transcriptional speeds” and priorities under identical dual CK supplies.

In summary, our data on global assessment of mRNA landscape and the functional annotation of filtered DEGs suggest that the synergistic effect of BA+KIN was achieved through two distinct strategies: cv. Húsvéti rozmaring underwent a rapid metabolic and structural reorganization of the photosynthetic system, while cv. McIntosh responded through a highly localized transcriptional reprogramming. These results provide a molecular basis for explaining the cultivar-specific CK effects in apple shoot multiplication.

Following the evaluation of the global mRNA transcriptome and the DEGs specifically filtered for the BA+KIN treatment, a targeted bioinformatic analysis was conducted on genes involved in hormonal biosynthesis, metabolism and signaling pathways. This approach enabled a more precise identification of the molecular drivers behind the observed morphological superiority.

The integration of morphological and transcriptomic data for the Húsvéti rozmaring cultivar revealed a clear molecular-to-phenotypic bridge, where the superior growth characteristics observed under BA+KIN treatment are directly underpinned by a profound reprogramming of the hormonal transcriptome. The high multiplication rate and maximal shoot length achieved with this dual CK synergy are reflected in the robust activation of auxin-responsive genes. This suggests that the combined application of BA+KIN did not merely stimulate cell division but potentially triggered a robust internal auxin signaling network that sustained vigorous biomass accumulation and fresh weight gains, which may help in preventing the stunted morphology often associated with high-dose single CK (BA) treatments [26].

The extreme up-regulation of primary response genes, particularly auxin-induced protein 15A-like and SAUR21-like DEGs, points toward a mobilization to drive rapid cellular changes. These SAUR proteins are thought to act as effectors that promote cell expansion and elongation by activating plasma membrane -ATPases, potentially supporting the superior shoot lengths observed [35]. The up-regulation of auxin-binding proteins; ABP19a and ABP20, suggests an increased capacity for auxin perception. These proteins promote rapid, non-genomic responses by stabilizing PIN transporters, thereby ensuring the maintenance of efficient polar auxin transport [36]. The induction of ARF18 transcription factor likely enables it to bind to specific auxin response elements, thereby regulating target gene expression, necessary for shoot proliferation and biomass accumulation [37]. While the induction of Aux/IAA repressors (e.g., IAA27, IAA3-like) suggests a necessary feedback loop to maintain homeostasis, influx (LAX1, LAX3) and efflux (PIN7) carriers are activated at the same time to ensure a highly active directional transport system. Specifically, the role of LAX3 in facilitating organ development through cell wall remodeling offers a plausible explanation for the cultivar’s ability to maintain high intracellular auxin flux, resulting in vigorous biomass gains (Table 2; Figure 3).

Furthermore, the significant down-regulation of GA receptors and oxidase genes provides a potential mechanistic explanation for the observed shift in shoot architecture. By suppressing GA-mediated apical dominance at the transcriptomic level, the plant appeared to reallocate resources toward the activation of multiple axillary buds, leading to the high multiplication rates documented [38,39,40]. This molecular antagonism, which seems precisely calibrated in cv. Húsvéti rozmaring, likely allows for high-frequency shoot proliferation without sacrificing the elongation quality necessary for successful shoot multiplication. This balanced development was probably primarily driven by the dramatic activation of the auxin response machinery. Additionally, the down-regulation of cytokinin riboside processing genes may indicate a homeostatic feedback loop, suggesting that the explant was successfully managing the high exogenous hormone load to maintain a stable, non-toxic physiological state [41]. Ultimately, these transcriptomic results suggest a regulatory “blueprint” for the optimal morphological vigor seen in this cultivar, supporting the hypothesis that the BA+KIN interaction acts as a master regulator of the apple’s developmental plasticity in vitro (Table 2; Figure 3).

In the context of the transcriptomic data for cv. Húsvéti rozmaring treated with BA+KIN, the JA and ABA pathways appear to function as critical secondary regulators of the plant’s in vitro developmental stability [42]. The differential expression of these pathways—characterized by the down-regulation of JA-induced and methyl jasmonate genes and the mixed regulation of ABA-related genes—points toward a significant suppression of stress-induced maturation and senescence. While CK and auxin drive the primary growth response, the transcriptomic evidence suggests that the BA+KIN treatment may actively repress JA signaling, which is known to antagonize CK-induced cell division and shoot elongation [43,44]. By down-regulating the JA pathway, the cultivar effectively potentially removes a major molecular “brake”, allowing for the rapid shoot multiplication and high biomass accumulation documented in the morphological results (Table 1 and Table 2; Figure 3).

The regulation of the ABA pathway further clarifies this developmental shift, as CK is a known antagonist of ABA-mediated growth inhibition [45]. The observed down-regulation of specific ABA-responsive genes reflects a molecular strategy to bypass the inhibitory effects of ABA on the cell cycle and stomatal opening, thereby maintaining the high metabolic activity required for in vitro vigor [46]. Conversely, the slight up-regulation of other ABA-related components may represent a homeostatic effort to maintain a baseline level of stress tolerance within the artificial culture environment without triggering full senescence. Together, these results indicate that the BA+KIN synergy functions beyond simple growth promotion, instead orchestrating a comprehensive “anti-stress” molecular profile. Rather than acting in isolation, this hormonal combination shifts the plant’s global transcriptomic state toward developmental stability by neutralizing inhibitory signals and fostering a resilient, high-vigor physiological environment. By simultaneously silencing the JA maturation signal and neutralizing ABA growth inhibitors, the treatment locks the cv. Húsvéti rozmaring explants into a high-performance juvenile state, which is the essential molecular prerequisite for the superior shoot quality and multiplication rates observed (Table 2; Figure 3).

The distinct morphological superiority of the BA+KIN treatment in cv. McIntosh—characterized by a peak multiplication rate of 5.98 and a three-fold increase in shoot fresh weight compared to the control—was strongly supported by the underlying transcriptomic profile, which reveals a complex orchestration of multi-hormone signaling pathways. While the morphological data confirmed that cv. McIntosh was less inherently vigorous in biomass accumulation than cv. Húsvéti rozmaring, the transcriptomic response to the BA+KIN combination suggests a high degree of hormonal plasticity specifically geared toward axillary bud activation and cellular differentiation (Table 2; Figure 3).

In the case of cv. McIntosh, the significant up-regulation of specific SAUR genes (SAUR50-like, SAUR12-like) and various Aux/IAA DEGs (IAA33, IAA28-like, IAA2-like) may indicate a rapid transcriptomic response to the BA+KIN treatment. In parallel with this, the up-regulation of two auxin efflux carrier components (PIN10 and efflux carrier component 1b) was observed, to possibly enhance the polar auxin system. As key regulators of polar auxin transport, PIN family members may contribute to auxin-mediated developmental and growth processes [47]. Overall, the simultaneous induction of small auxin up-regulated RNAs (SAURs), repressors (Aux/IAA), and auxin efflux carriers indicates comprehensive activation of the auxin regulatory network in this cultivar (Table 2; Figure 3).

Furthermore, the significant expression of CK metabolic genes, such as cytokinin hydroxylase-like and cytokinin dehydrogenase 1-like, suggests a highly active homeostatic mechanism in cv. McIntosh. The up-regulation of hydroxylase-like proteins specifically in response to BA-based treatments is a known marker for trans-zeatin biosynthesis, which further promotes cell division and shoot quality (Table 2; Figure 3). This was reflected morphologically in the treatment’s ability to maintain high shoot length (18.9 mm) despite the high multiplication rate, avoiding the stunting often associated with high BA concentrations alone (Table 1) [48,49].

The transcriptomic profile also pointed toward a secondary role for ABA and GA pathways in modulating growth. The down-regulation of gibberellin 20-oxidase-like and the up-regulation of ABA-insensitive proteins (e.g., PYR) suggest that the BA+KIN treatment may simultaneously suppress elongation-favoring GA pathways while sensitizing tissues to senescence-related triggers [50]. In cv. McIntosh, this results in a compact but highly proliferative growth habit that is unique among the studied cultivars. Ultimately, these results demonstrate that the BA+KIN combination provided an optimal transcriptomic landscape for the shoot multiplication of cv. McIntosh (Table 2; Figure 3) [51].

The morphological and transcriptomic data of the inter-cultivar comparisons together suggest that the superior regenerative capacity of the Húsvéti rozmaring cultivar under BA+KIN treatment was driven by a more efficient utilization of exogenous hormones rather than an increase in endogenous biosynthesis. Morphologically, the combination of BA+KIN yielded the highest multiplication rates and shoot fresh weights in both cultivars; however, cv. Húsvéti rozmaring significantly outperformed cv. McIntosh in shoot length (SL), achieving 27.95 mm compared to cv. McIntosh’s 18.9 mm (Table 1). This phenotypic divergence in growth vigor occurred despite a profound and systemic down-regulation of hormone-related genes in cv. Húsvéti rozmaring relative to cv. McIntosh. The transcriptomic profile of cv. Húsvéti rozmaring showed a massive suppression of genes involved in the biosynthesis and signaling of gibberellins (e.g., GA20ox2), auxins (e.g., IAA1-like), and CKs (Table 2; Figure 3).

This apparent paradox, where the faster-growing cultivar exhibits lower transcript levels for growth-promoting pathways, likely indicates a state of high metabolic efficiency or a feedback inhibition mechanism [52,53]. In cv. Húsvéti rozmaring, the exogenous BA and KIN appeared to satisfy the physiological requirements for growth more effectively, triggering a compensatory down-regulation of endogenous pathways to maintain hormonal homeostasis. In the cv. Húsvéti rozmaring vs. cv. McIntosh comparison, all nine DEGs were significantly down-regulated. This suggests that cv. Húsvéti rozmaring is considerably more sensitive to the antagonistic effects of exogenous CKs on auxin-related pathways. For example, the pronounced down-regulation of IAA1-like gene and several auxin efflux carriers (auxin efflux carrier component 1c, auxin efflux carrier component 1b, and auxin efflux carrier component 2-like) suggests that cv. Húsvéti rozmaring may require reduced internal auxin transport to achieve enhanced shoot elongation. Conversely, the higher transcript levels in cv. McIntosh may reflect a “starvation” response or a less efficient perception of the BA+KIN treatment, where the plant over-activates endogenous machinery to compensate for a weaker morphological response (Table 1 and Table 2; Figure 3). Ultimately, the Húsvéti rozmaring cultivar’s ability to maximize biomass and shoot elongation while maintaining a suppressed endogenous hormonal transcriptome suggests a more streamlined and responsive signaling network, making it better suited for micropropagation under these specific CK conditions.

## 4. Materials and Methods

### 4.1. Plant Material and In Vitro Maintenance Conditions

In vitro shoot cultures of two *Malus* × *domestica* Borkh. cultivars, Húsvéti rozmaring and McIntosh, were utilized as the primary explant sources. The stock cultures were maintained on a basal MS medium [54] supplemented with 0.49 μM IBA, 2.22 μM BA, and 0.58 μM GA3. The medium contained 3% (*w*/*v*) sucrose and was solidified with 0.7% (*w*/*v*) agar, with the pH adjusted to 5.8 prior to sterilization (121 °C, 1.2 bar, 20 min). Cultivation took place in a controlled environment at 23 ± 2 °C under a 16/8 h photoperiod, with a light intensity of 106 µmol s^−1^ m^−2^ provided by a 1:1 mixture of daylight and warm white fluorescent lamps. To ensure a uniform physiological state and to eliminate potential carry-over effects from previous CK exposure, a final 4-week subculture was performed on a CK-free medium prior to explant isolation. Explants were collected at the end of this standardized subculture period, thereby minimizing variability associated with developmental stage.

### 4.2. Experimental Cytokinin Treatments

Following the CK-free subculture, 20 mm shoot segments were isolated from in vitro mother shoots and transferred to experimental media (five explants per vessel, 70 mL medium). The basal composition was identical to the maintenance medium, varying only in CK supplementation. In both single and dual treatments, the CK concentrations were selected based on established literature standards to evaluate their specific physiological effects, rather than maintaining a constant total molar concentration: (1) CK-free control (NCK), (2) 4.5 μM BA, (3) 4.5 μM KIN, and (4) a dual CK treatment of 4.5 μM BA combined with 7.0 μM KIN. The selected CK concentrations are within the range commonly used for in vitro shoot propagation of apple and other woody plants, representing biologically effective and optimal concentration levels [3,4,26,27]. However, in the case of the combination treatment, the KIN concentration of 7.0 μM was also use to promote the potential synergistic interaction between the two CKs, thereby reducing the dominance of BA [4]. Environmental conditions were kept identical to those used during maintenance.

### 4.3. Data Collection and Statistical Analysis of Shoot Multiplication Experiments

After a 4-week incubation period, shoot fresh weight (SFW, g/vessel), multiplication rate (SN, number of new shoots per explant), and shoot length (SL, mm) were recorded from ten replicates per treatment (ten vessels, each vessel contained 5 shoot explants). Each vessel was treated as an independent experimental unit.

Morphological parameters were processed using IBM SPSS Statistics v21.0. To evaluate the influence of cultivar and CK type, univariate Analysis of Variance (ANOVA) was performed. Differences between specific treatment means were determined using one-way ANOVA followed by Tukey’s post hoc test, with a significance threshold set at *p* < 0.05.

### 4.4. Sample Collection and Isolation of mRNA

At the end of the subculture, apple shoots were collected from three independent biological replicates for each treatment (NCK, BA, KIN, and BA+KIN) and for both cultivars. The samples were immediately flash-frozen in liquid nitrogen and stored at −80 °C for subsequent RNA isolation.

Total RNA was isolated in triplicate for each treatment using the Zymo Research Quick-RNA™ Plant Miniprep kit (Zymo Research, Irvine, CA, USA) following the instruction manual. The quantity and quality of the purified RNA samples were assessed by spectrophotometry on an Implen N50 Nanophotometer (Implen, Munich, Germany) and by microfluidic electrophoresis using the Agilent 2100 Bioanalyzer (Agilent, Santa Clara, CA, USA) with an RNA 6000 Nano Kit (Agilent, Santa Clara, CA, USA) to ensure sample suitability for downstream applications.

### 4.5. mRNA Sequencing, Bioinformatic Analysis, and Functional Annotation of the Dataset

Following rigorous quality control, poly-A mRNA enrichment, cDNA library construction, and subsequent sequencing were performed by Novogene Co., Ltd. Libraries were sequenced on the Illumina NovaSeq X Plus platform, generating 150 bp paired-end reads (PE150).

Raw sequencing reads were evaluated for quality using FastQC (v0.12.1) [55] and processed with fastp (v0.26.0) [56] to remove adapter sequences and low-quality data. The resulting clean reads were aligned to the *Malus domestica* reference genome assembly GDT2T_hap1 (GCF_042453785.1) utilizing HISAT2 (v2.1.1) [57]. Read counts per gene were quantified using featureCounts (v2.1.1) [58].

Differential expression analysis was conducted using the DESeq2 package (v1.44.0) [59]. Genes meeting the thresholds of an adjusted *p*-value < 0.05 and a |log_2_ fold change| > 2 were considered as differentially expressed genes (DEGs). Functional enrichment analysis of DEGs for Gene Ontology (GO) terms and Kyoto Encyclopedia of Genes and Genomes (KEGG) pathways was executed using the g:Profiler2 package (v0.2.4) [60] applying a significance threshold of adjusted *p*-value < 0.05. All statistical analyses and data visualizations were performed within the R statistical computing environment (v4.4.1) [61], primarily utilizing the ggplot2 package (v3.5.2) [62].

### 4.6. RT-qPCR Analysis for Validation

Following the protocols mentioned in the “Sample collection and isolation of mRNA” section, total RNA was purified and assessed. For cDNA synthesis, 200 ng/µL of mRNA was used as a template with the Reverse Transcriptase Core Kit (Eurogentec, Searing, Belgium) and 2.5 µM random nonamers. The reaction conditions were 25 °C (10 min), 48 °C (30 min), and 95 °C (5 min). All cDNA products were stored at −20 °C until use.

Two candidate housekeeping genes, actin (LOC103445585) and glyceraldehyde-3-phosphate dehydrogenase (GAPDH, LOC103403121), were evaluated for expression stability. Assessment via cycle quantification (Cq) [63] values, geNorm [64], NormFinder [65], and BestKeeper [66] algorithms—integrated through the RefFinder platform [67]—confirmed their suitability for normalization in this experimental context (Table S3).

For each of the three treatments within both cultivars, two representative candidate genes were selected from the RNA-seq data: one exhibiting a significant positive logarithmic fold change (LFC) and one exhibiting a significant negative LFC. The specific primers locus IDs and sequences for these treatment-specific targets are detailed in Table S3. Note that in the case of cv. McIntosh for the BA treatment group, there was no significantly down-regulated DEG (using a cutoff value of 2 for LFCs).

Gene sequences obtained from NCBI were used to design primers via SnapGene (v8.0) and checked by PCR Primer Stats (www.bioinformatics.org, accessed on 15 January 2026). The design criteria included a Tm of 56–62 °C, GC content of 40–60%, and a product length of 150–250 bp. To confirm primer specificity, BLASTn (Basic Local Alignment Sequence Tool for nucleotides) searches were performed against the nucleotide database to ensure no significant homology with non-target sequences.

Quantitative PCR was conducted using the Takyon™ No Rox^®^ SYBR MasterMix dTTP Blue kit (Eurogentec, Seraing, Belgium) according to the manufacturer’s instructions. Each 20 µL reaction contained 2.5 µL of cDNA template, 10 µL of Takyon™ MasterMix, and 0.1 µM of each forward and reverse primer. The thermal cycling profile initiated with a 3 min activation step at 95 °C, followed by 40 cycles of denaturation at 95 °C (10 s), primer-specific annealing at 54 °C (20 s), and extension at 72 °C (30 s). To ensure analytical precision, three technical replicates were performed for each of the three biological replicates.

Relative expression levels were determined using the 2^−ΔΔCq^ method [68], with calculations based on the mean of three technical replicates per biological sample. LFC values and their associated standard deviations were calculated in Microsoft Excel (Redmond, WA, USA). To assess the consistency between RNA-seq and RT-qPCR results, Pearson correlation coefficients were determined using the SRplot platform (https://www.bioinformatics.com.cn/en, accessed on 18 February 2026) [69]. Detailed correlation data and expression values are provided in Table S3.

## 5. Conclusions

The integration of morphological and transcriptomic data revealed that while both apple cultivars achieved their peak performance under the BA+KIN synergistic treatment, they utilized distinct molecular strategies to reach this high-performance state. For cv. Húsvéti rozmaring, the superior shoot length and biomass were the result of a streamlined signaling network. By systematically down-regulating endogenous biosynthetic pathways (auxin, GA, and JA), cv. Húsvéti rozmaring achieved a state of high metabolic efficiency, using exogenous CKs to maintain a “high-performance juvenile state” that bypassed typical stress-induced growth inhibitors.

In contrast, cv. McIntosh exhibited a more reactive transcriptomic profile, characterized by a massive up-regulation of auxin signaling and complex homeostatic feedback loops. Its success under BA+KIN was rooted in hormonal plasticity, where high-intensity crosstalk between auxin and CK facilitated aggressive axillary bud release, resulting in the highest recorded multiplication rates despite a more compact growth habit.

Ultimately, this study confirmed that the BA+KIN combination acted as a master regulator of in vitro developmental plasticity in apple. While the specific transcriptomic “blueprints” differ, with cv. Húsvéti rozmaring favoring elongation through signaling efficiency and cv. McIntosh favoring proliferation through intense hormonal crosstalk, both cultivars converged on a shared physiological outcome: the successful management of high exogenous hormone loads to achieve optimal shoot quality and multiplication. These findings provide a molecular foundation for refining cultivar-specific micropropagation protocols in fruit trees.

Our findings underscore that the transcriptome serves as a predictive roadmap for downstream physiological changes, suggesting that future research incorporating multiple sampling time points and a broader range of genotypes will be essential to fully resolve these regulatory processes. Validating these transcriptomic ‘blueprints’ through endogenous hormone quantification, protein expression analyses, and metabolic profiling will be instrumental in developing targeted, genotype-specific ‘smart’ micropropagation protocols for elite fruit tree cultivars.

## Figures and Tables

**Table 1:** Growth and development of in vitro apple shoots on media with different cytokinin content.

CK Content of the Medium	Multiplication Rate (SN/Explant)	SL/Shoot (mm)	SFW (g/Vessel)
**cv. McIntosh**			
**CK-free**	1.29 ± 0.07 c	17.9 ± 6.6 a	0.51 ± 0.28 c
**BA**	4.96 ± 1.93 b *	15.9 ± 6.6 b *	1.22 ± 0.93 b *
**KIN**	1.04 ± 0.20 c	19.8 ± 3.9 a	0.43 ± 0.21 c
**BA+KIN**	5.98 ± 2.53 a	18.9 ± 5.7 a *	1.55 ± 0.82 a *
**cv. Húsvéti Rozmaring**			
**CK-free**	1.53 ± 0.16 c	15.91 ± 0.95 c	0.55 ± 0.05 c
**BA**	3.58 ± 0.21 b *	26.54 ± 0.90 ab *	1.73 ± 0.09 b *
**KIN**	1.07 ± 0.04 c	22.88 ± 0.90 b	0.61 ± 0.04 c
**BA+KIN**	4.90 ± 0.37 a	27.95 ± 1.41 a *	2.22 ± 0.15 a *

Mean values (±standard error) followed by different letters in each column indicate significantly (*p* < 0.05) different values between treatments, while mean values followed by * indicate the significantly (*p* < 0.05) different values between cultivars at the same CK supply. Significance marks (letters or asterisk) are indicated according to the ANOVA followed by Tukey’s test in the case of variance equality, or to the Welch ANOVA followed by Games-Howell test in the case of non-equality of variances. Abbreviations: BA: 6-benzyladenine; CK: cytokinin; KIN: kinetin; SN: shoot number; SL: shoot length; SFW: shoot fresh weight.

**Table 2:** Number of significantly differentially up-, or down-regulated genes (DEGs) according to their encoded products (proteins or enzymes) in response to dual cytokinin (BA+KIN) treatment after comparative filtering and excluding DEGs that were present in the individual BA vs. NCK and KIN vs. NCK comparisons in cvs. Húsvéti rozmaring (HR) and McIntosh (MI), as well as between cultivars (BTWC). Blue and red arrows indicate down- and up-regulation, respectively.

Enzymes and Proteins			HR	MI	BTWC
**Cytokinin metabolic enzymes**					
	***Cytokinin activating enzymes***				
		cytokinin riboside 5′-monophosphate phosphoribohydrolase LOG1-like	1 ↓		
		cytokinin riboside 5′-monophosphate phosphoribohydrolase LOG7			1 ↓
		cytokinin riboside 5′-monophosphate phosphoribohydrolase LOG8-like			1 ↓
	***Side-chain modifying enzymes***				
		cytokinin hydroxylase-like		1 ↑	2 ↓
	***Cytokinin oxidases/dehydroxygenases***				
		cytokinin dehydrogenase 1-like		1 ↑	
		cytokinin dehydrogenase 3-like			1 ↓
**Proteins included in the gibberellic acid (GA) signaling pathway**					
	***GA biosynthesis enzymes***				
		gibberellin 20 oxidase 2			1 ↓
		gibberellin 20-oxidase-like		1 ↓	
	***GA catabolism enzymes***				
		gibberellin 3-beta-dioxygenase 1-like		1 ↑	1 ↓
		gibberellin 2-beta-dioxygenase 2-like			1 ↓
		gibberellin 2-beta-dioxygenase 8	1 ↓		2 ↓
		gibberellin 2-beta-dioxygenase 1-like		1 ↓	
	***GA signaling and perception***				
		gibberellin receptor GID1B-like	1 ↓		
	***Downstream responsive proteins***				
		gibberellin-regulated protein 4		1 ↑	
		chitin-inducible gibberellin-responsive protein 1-like		1 ↓	
**Proteins included in the abscisic acid (ABA) pathway**					
	***Hormone perception***				
		abscisic acid receptor PYL3	2 ↑		
		abscisic acid receptor PYR1-like		1 ↑	
	***Hormone catabolism***				
		abscisic acid 8′-hydroxylase CYP707A2-like	1 ↓	1 ↓	
		abscisic acid 8′-hydroxylase CYP707A2	1 ↓		1 ↓
		abscisic acid 8′-hydroxylase 4		1 ↑	
	***Downstream signaling***				
		ABSCISIC ACID-INSENSITIVE 5-like protein 5		1 ↑	
**Auxin signaling pathway (regulatory proteins and transporters)**					
	***Nuclear signaling: repressors***				
		auxin-responsive protein IAA1-like	1 ↓		1 ↓
		auxin-responsive protein IAA2-like		1 ↑	
		auxin-responsive protein IAA3-like	1 ↑		
		auxin-responsive protein IAA11-like		1 ↓	
		auxin-responsive protein IAA13		1 ↑	
		auxin-responsive protein IAA16	1 ↑		
		auxin-responsive protein IAA21		1 ↑	
		auxin-responsive protein IAA26		1 ↑	
		auxin-responsive protein IAA26-like		1 ↑	
		auxin-responsive protein IAA27	1 ↑	1 ↑	
		auxin-responsive protein IAA28-like		1 ↑	
		auxin-responsive protein IAA33		1 ↑	1 ↓
		auxin-repressed 12.5 kDa protein		2 ↓	
	***Nuclear signaling: activators and effectors***				
		auxin response factor 18	1 ↑		
		auxin response factor 2-like			1 ↓
		auxin-responsive protein SAUR21-like	2 ↑		
		auxin-responsive protein SAUR50-like		2 ↑	
		auxin-responsive protein SAUR76-like			1 ↓
		protein SMALL AUXIN UP-REGULATED RNA 12		1 ↑	
		protein SMALL AUXIN UP-REGULATED RNA 12-like	1 ↑	3 ↑	
		protein SMALL AUXIN UP-REGULATED RNA 51		1 ↑	
		auxin-induced protein 15A-like	5 ↑		
		auxin-induced protein 22D			1 ↓
		auxin-induced protein 22D-like	1 ↑		
	***Auxin transport***				
		auxin transporter-like protein 2	1 ↑		
		auxin transporter-like protein 3	1 ↑		
		auxin efflux carrier component 3	1 ↑		
		probable auxin efflux carrier component 1b		1 ↑	1 ↓
		probable auxin efflux carrier component 1c		1 ↑	2 ↓
		auxin efflux carrier component 2-like			1 ↓
	***Auxin perception***				
		auxin-binding protein ABP19a	1 ↑		
		auxin-binding protein ABP20	1 ↑		
**Jasmonic acid (JA) signaling pathway**					
	***Activators/converters***				
		methyl jasmonate esterase 1-like	1 ↓		3 ↓
	***Deactivators***				
		jasmonate-induced oxygenase 1-like	1 ↓		1 ↓
**Brassinosteroid signaling pathway**					
	***Ubiquitin-mediated regulation***				
		brassinosteroid-responsive RING protein 1-like	1 ↑		
	***Cell wall remodeling***				
		brassinosteroid-regulated protein BRU1-like		1 ↓	

## Data Availability

The transcriptomics data has been deposited at the NCBI SRA database (https://www.ncbi.nlm.nih.gov/sra; accessed on 20 February 2026), where it is available under the BioProject identifier PRJNA1358718. Apart from that, all relevant data can be found within the manuscript and its Supplementary Materials.

## Supplementary Materials

The following supporting information can be downloaded at: https://www.mdpi.com/article/10.3390/plants15071001/s1, Figure S1. Volcano plots on total number of expressed genes in in vitro apple shoots in comparisons between (A) BA vs. control (NCK), (B) KIN vs. control, and (C) BA+KIN vs. control, and total number of significantly differentially expressed, up- and down-regulated genes in the same comparisons, 4 weeks after in vitro cultivation of shoots. Volcano plots were generated by ggplot2 R package; *x*-axis representing the log_2_ fold change and *y*-axis the adjusted *p*-values of the genes. The blue dots indicate down-regulated gene expression, the red dots indicate up-regulated gene expression, and the gray dots indicate the gene expression without significant differences, in each comparison; Figure S2. Functional distribution of DEGs in response to dual cytokinin (BA+KIN) treatment across GO categories. GO enrichment dot plots based on significantly up- and down-regulated genes enriched in biological processes, cellular components, molecular functions and KEGG pathways; Table S1. All significantly up- and down-regulated genes (DEGs) at cutoff value of LFC ≥ 2, and based on mRNA-seq of 51,804 genes (promoters and coding regions) in comparisons of BA vs. control (NCK), KIN vs. control, and BA+KIN vs. control, respectively, in cvs. Húsvéti rozmaring and McIntosh, and between cultivars, 4 weeks after cytokinin treatments applied in the shoot multiplication medium of in vitro apple shoots. In addition, DEGs in BA+KIN vs. control comparisons in both cultivars and between cultivars, respectively, after comparative filtering and excluding DEGs that were present in the individual BA vs. NCK and KIN vs. NCK comparisons; Table S2. GO enrichment for all, significantly up- and down-regulated biological processes, cellular components, molecular functions and KEGG pathways in response to BA+KIN compared to control after filtering out differentially expressed genes (DEGs) responding to single cytokinin (BA or KIN) application in. cvs. Húsvéti rozmaring and McIntosh and between cultivars comparisons, respectively. Up- and down-regulated genes are listed separately, in separate worksheets; Table S3. Validation of mRNA-seq data by RT-qPCR. For each treatment and cultivar combination, two representative genes (one up-regulated and one down-regulated) were selected for mRNA-seq validation. The table summarizes the locus IDs, primer sequences, and a comparison between RNA-seq log_2_ fold change (LFC) and qPCR relative expression levels. Housekeeping genes were selected. The Pearson correlation coefficient (R) was provided to indicate the degree of concordance between the two platforms across the experimental conditions.

## Abbreviations

The following abbreviations are used in this manuscript:

ABA	abscisic acid
ABP	auxin-binding protein
ACTIN	actin (housekeepeng gene)
ANOVA	analysis of variance
AIP15A-like	auxin-induced protein 15A-like
ARF	auxin response factor
Aux/IAA	auxin-responsive protein family (Aux/IAA repressors)
BA	6-benzyladenine
BLASTn	Basic Local Alignment Sequence Tool for nucleotides
BTWC	between cultivars comparison
BRU1	brassinosteroid-regulated protein
cDNA	complementary DNA
CK	cytokinin
DEG	differentially expressed gene
GA	gibberellic acid
GAPDH	glyceraldehyde-3-phosphate dehydrogenase
GID1	gibberellin receptor GID1 (gibberellin insensitive dwarf1)
GO	Gene Ontology
HR	cv. Húsvéti rozmaring
IBA	indole-3-butyric acid
JA	jasmonic acid
KEGG	Kyoto Encyclopedia of Genes and Genomes
KIN	kinetin
LAX	auxin influx carrier
LFC	logarithmic fold change
MI	cv. McIntosh
mRNA	messenger RNA
MS medium	Murashige and Skoog medium
NCK	cytokinin-free control
PCA	Principal Component Analysis
PGRs	plant growth regulators
PIN	auxin efflux carrier
RNA-seq	RNA sequencing
RT-qPCR	reverse transcription quantitative polymerase chain reaction
SAUR	small auxin up-regulated RNA
SL	shoot length
SN	shoot number
SFW	shoot fresh weight
