# Supplementary material for: Comparative Transcriptomic Analysis of Two Apple Cultivars in Response to Dual Cytokinin Applied In Vitro
Source: Plants (Basel). 2026 Mar 25;15(7):1001. doi: 10.3390/plants15071001 (PMC13074517; doi:10.3390/plants15071001)

cv. Húsvéti rozmaring

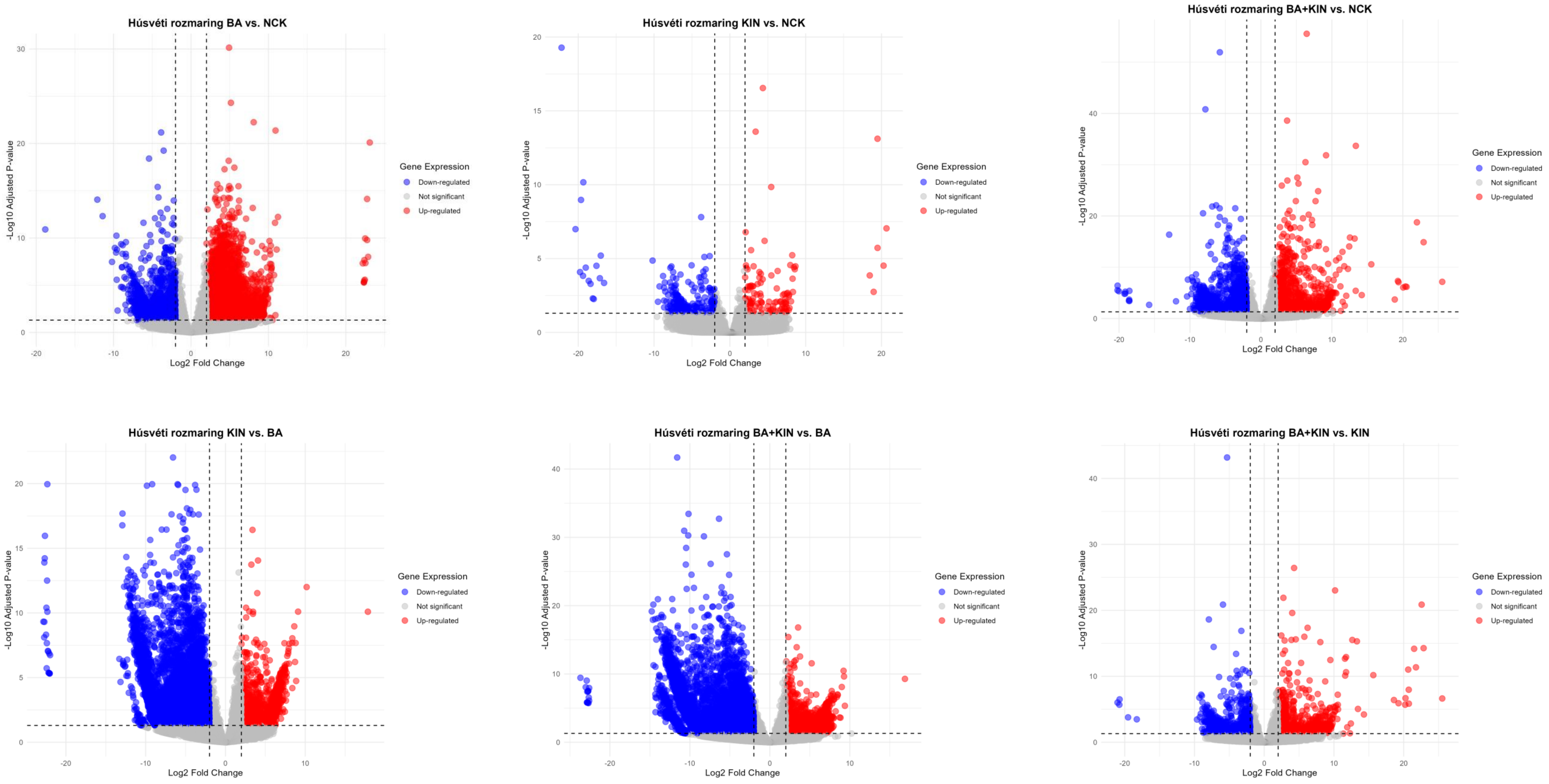

cv. McIntosh

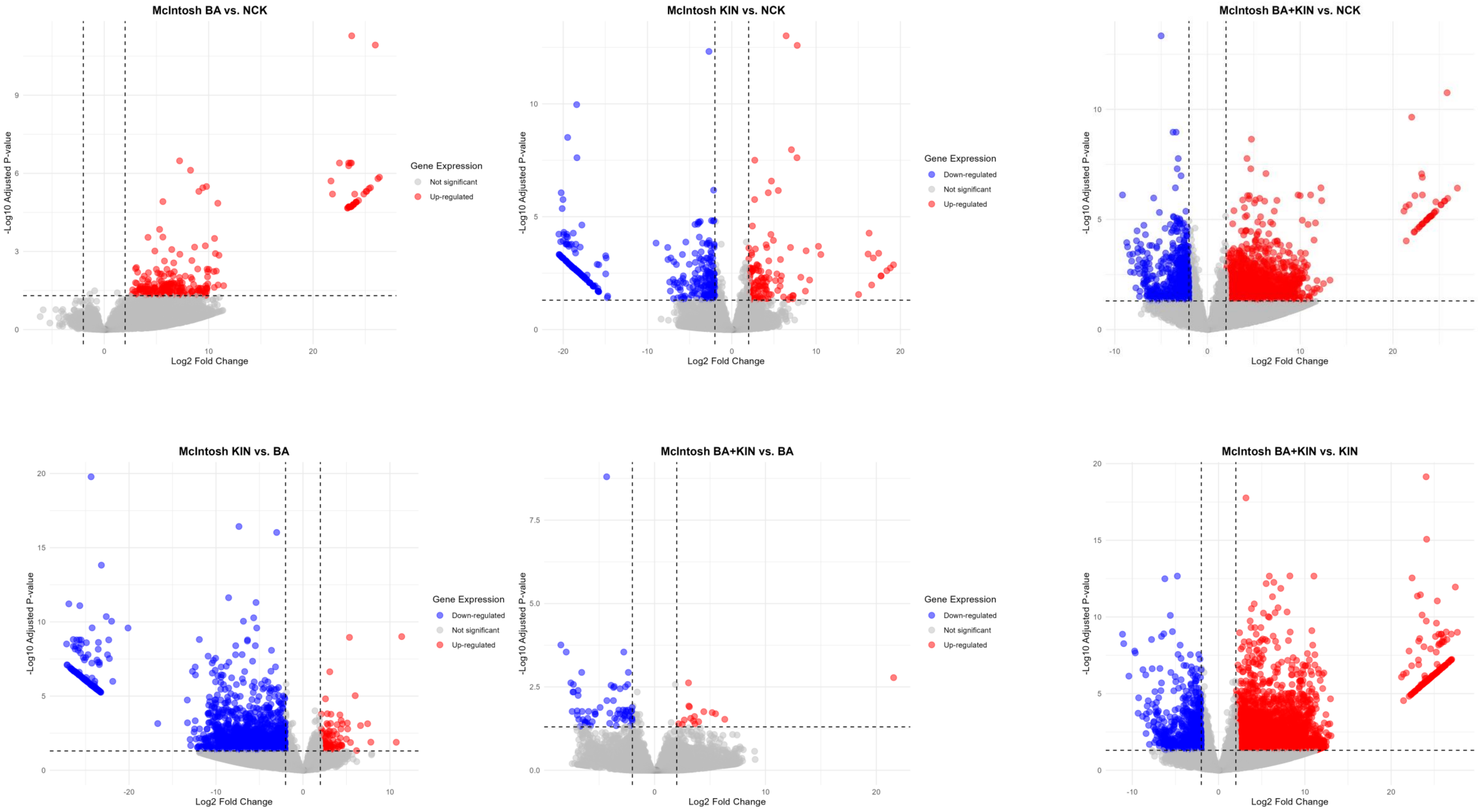
McIntosh BA vs. NCK
McIntosh KIN vs. NCK
McIntosh BA+KIN vs. NCK
McIntosh KIN vs. BA
McIntosh BA+KIN vs. BA
McIntosh BA+KIN vs. KIN
-Log10 Adjusted P-value
Log2 Fold Change
Gene Expression
Down-regulated
Not significant
Up-regulated

Between cultivars comparisons

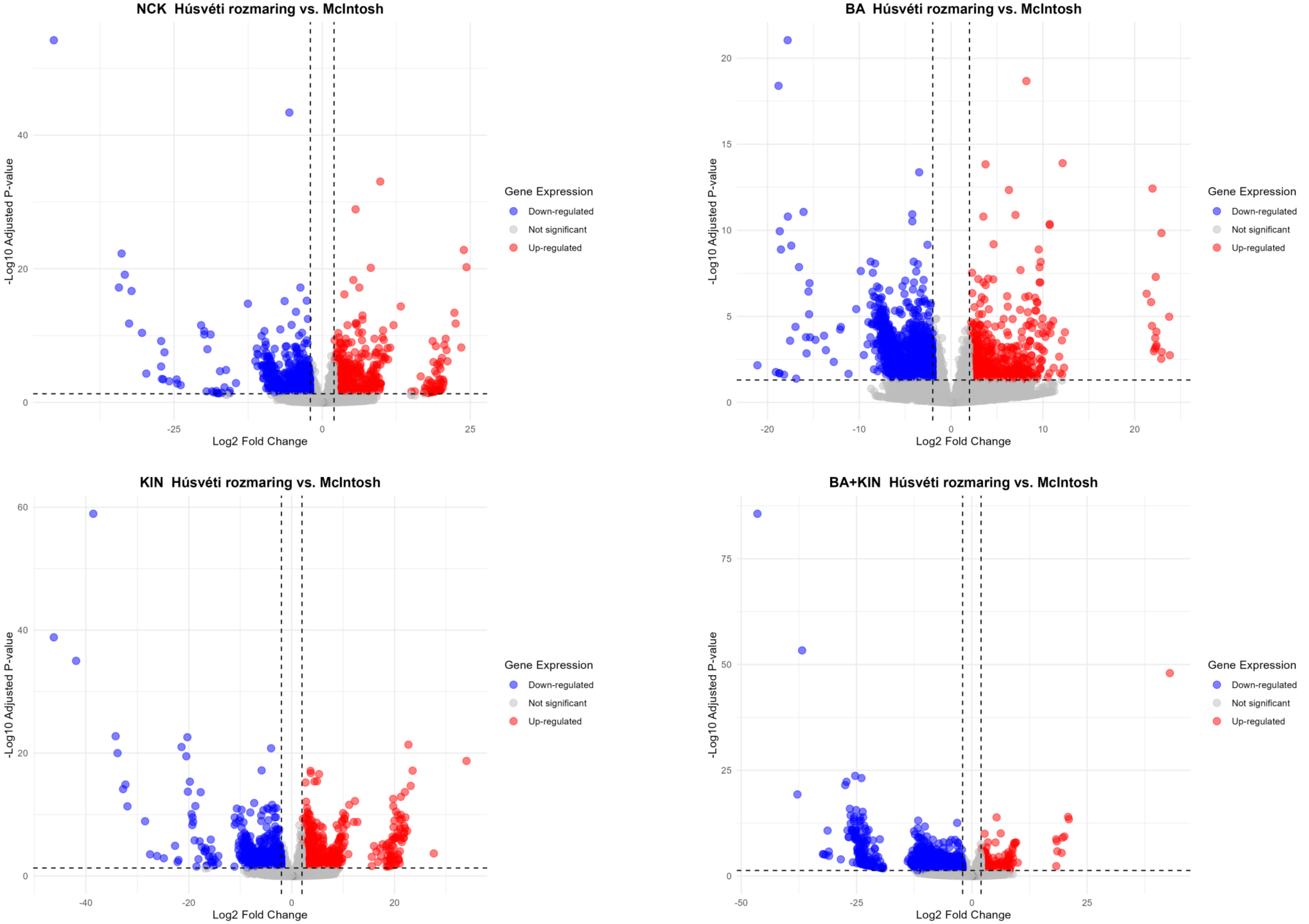

Supplement: Supplementary file 1 [file plants-15-01001-s001.zip › Figure S1.pdf]
