# Supplementary material for: Comparative Transcriptomic Analysis of Two Apple Cultivars in Response to Dual Cytokinin Applied In Vitro
Source: Plants (Basel). 2026 Mar 25;15(7):1001. doi: 10.3390/plants15071001 (PMC13074517; doi:10.3390/plants15071001)
Supplement: Supplementary file 1 [file plants-15-01001-s001.zip › Figure S2.pdf]

cv. Húsvéti rozmaring

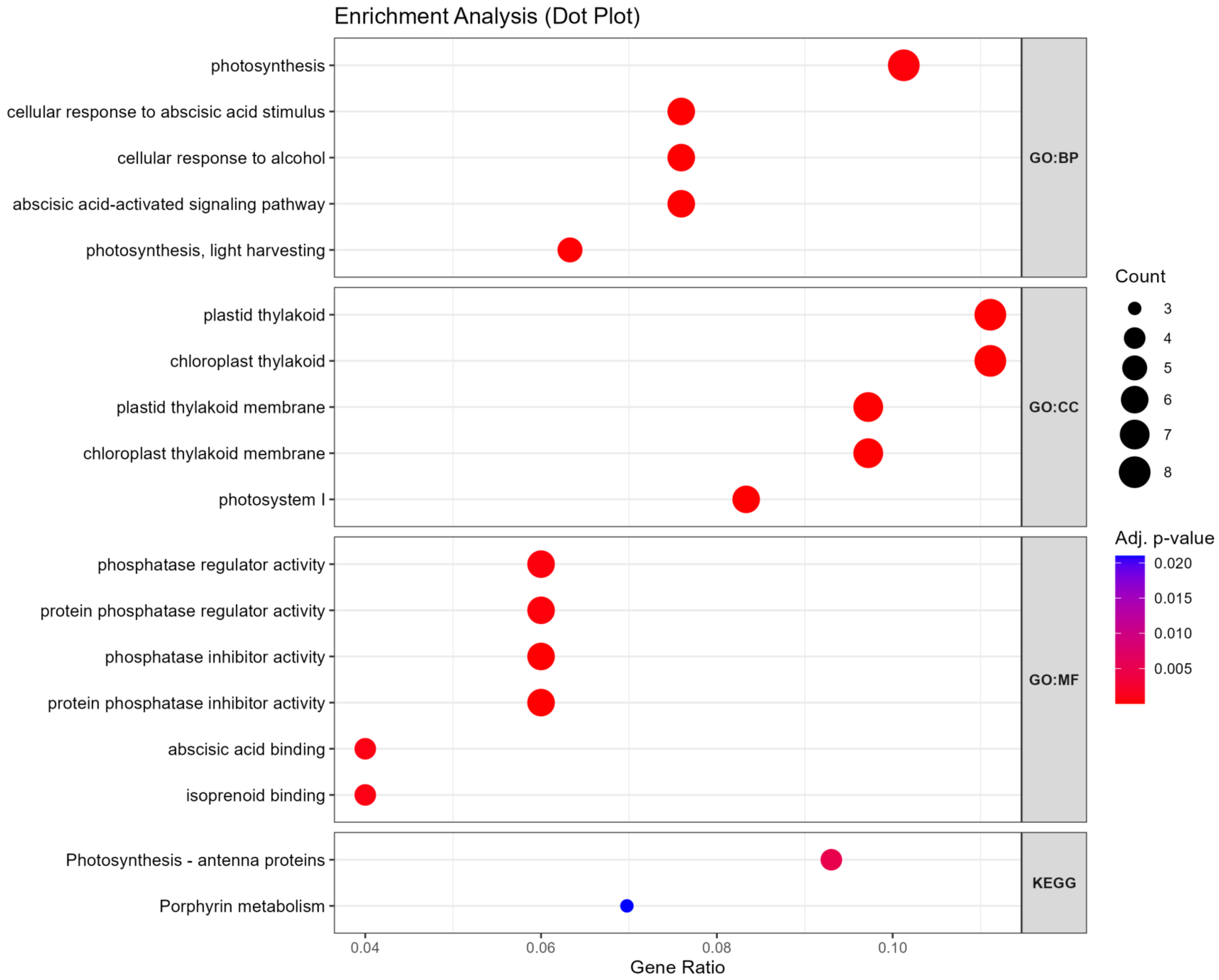

cv. Mc Intosh

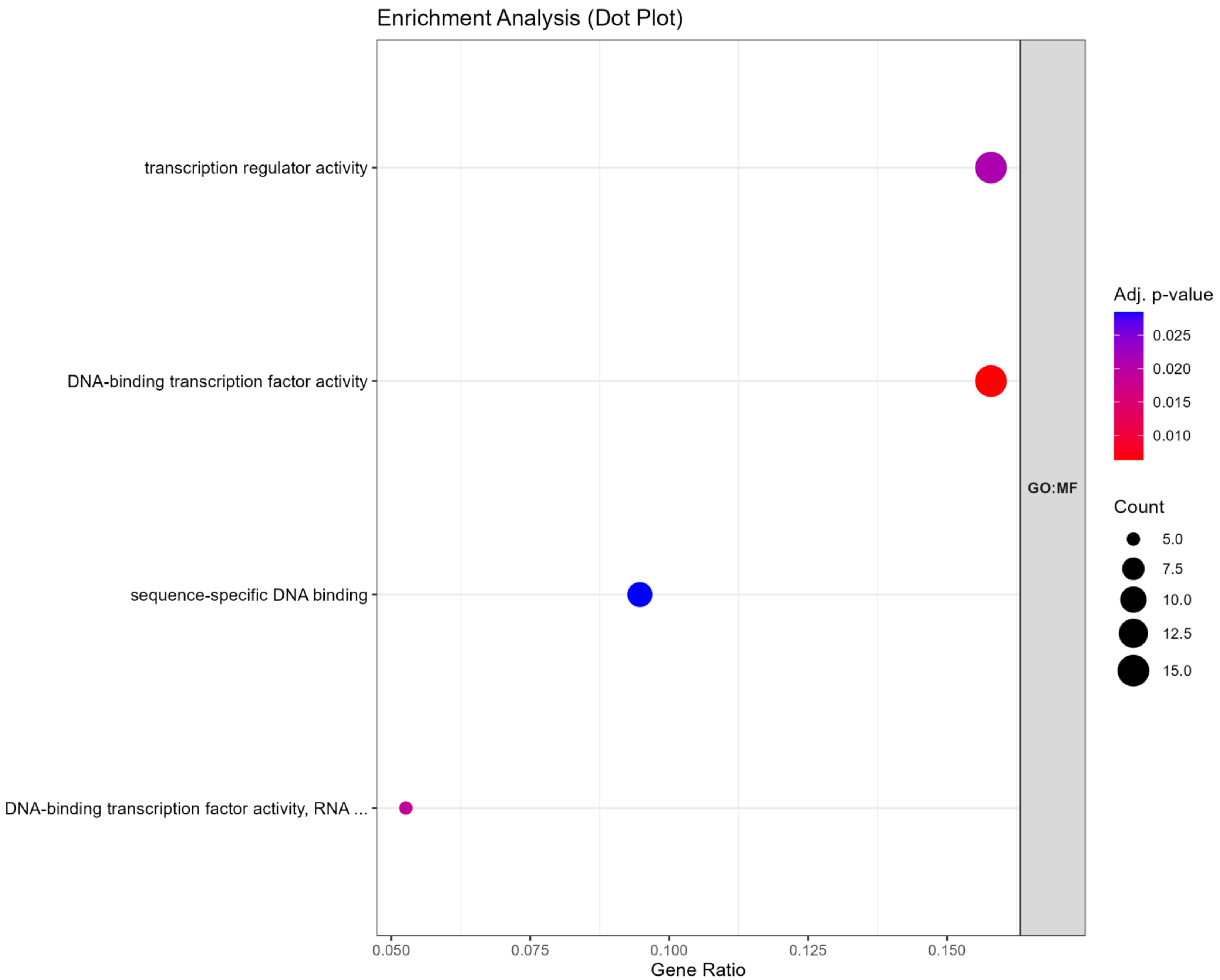
Enrichment Analysis (Dot Plot)
transcription regulator activity
DNA-binding transcription factor activity
sequence-specific DNA binding
DNA-binding transcription factor activity, RNA ...
GO:MF
0.050
0.075
0.100
0.125
0.150
Gene Ratio
Adj. p-value
0.025
0.020
0.015
0.010
Count
5.0
7.5
10.0
12.5
15.0

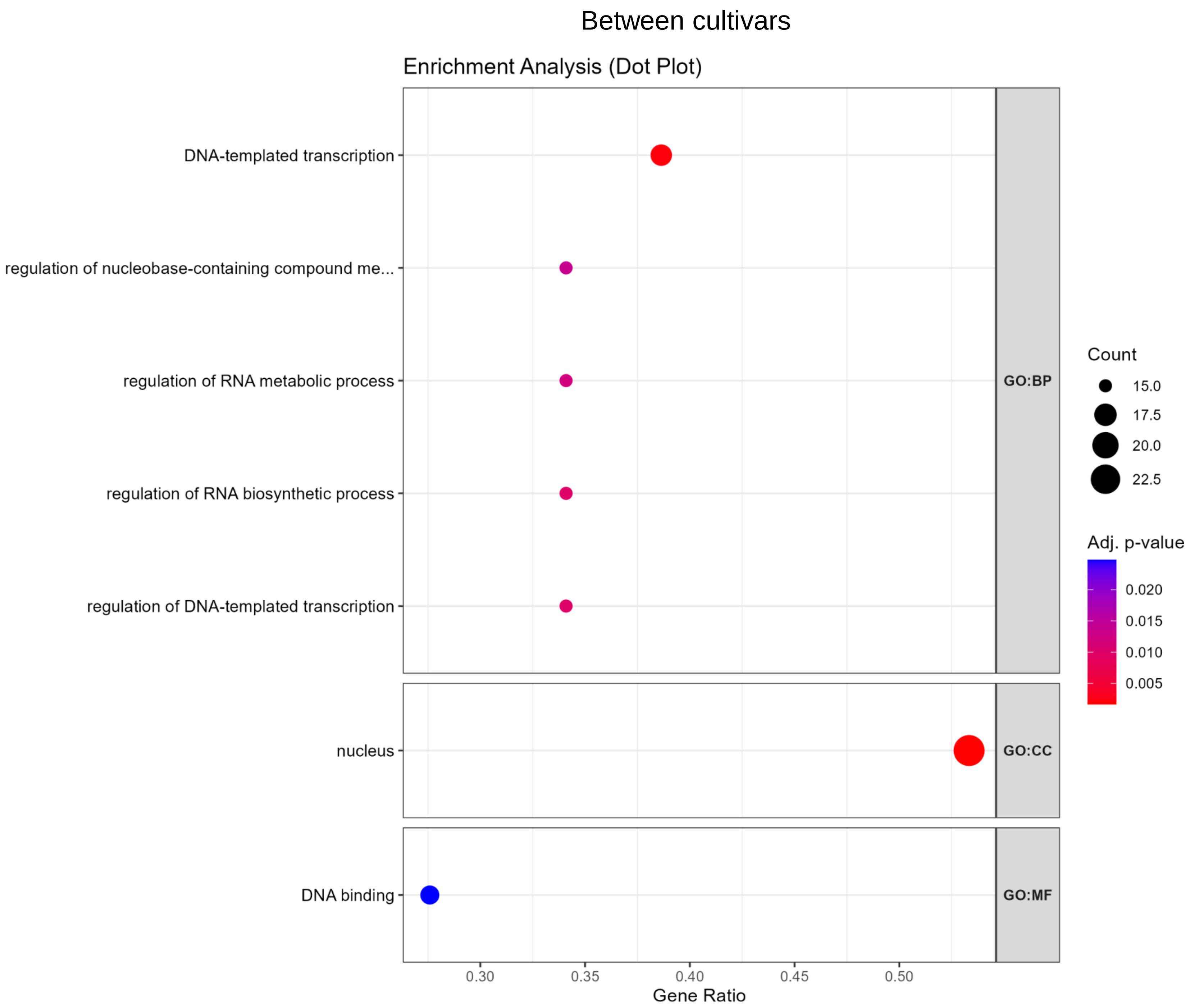
Between cultivars
Enrichment Analysis (Dot Plot)
DNA-templated transcription
regulation of nucleobase-containing compound me...
regulation of RNA metabolic process
regulation of RNA biosynthetic process
regulation of DNA-templated transcription
nucleus
DNA binding
GO:BP
GO:CC
GO:MF
Count
15.0
17.5
20.0
22.5
Adj. p-value
0.020
0.015
0.010
0.005
0.30
0.35
0.40
0.45
0.50
Gene Ratio
